# Representations of COVID-19: the pandemic in the context of international commuting migration from mining

**DOI:** 10.1590/1980-220X-REEUSP-2022-0382en

**Published:** 2023-11-17

**Authors:** Lise Maria Carvalho Mendes, Antonio Sabino da Silva, Nayara Gonçalves Barbosa, Larissa de Freitas Cardoso, Rosemary Ferreira de Andrade, Flávia Azevedo Gomes-Sponholz

**Affiliations:** 1 Universidade de São Paulo, Programa de Pós-Graduação em Enfermagem em Saúde Pública, Ribeirão Preto, SP, Brazil.; 2 Universidade Federal do Amapá, Programa de Pós-Graduação em Direito, Macapá, AP, Brazil.; 3 Universidade Federal de Juiz de Fora, Departamento de Enfermagem Materno-Infantil e Saúde Pública, Juiz de Fora, MG, Brazil.; 4 Universidade Federal do Amapá, Programa de Pós-Graduação em Ciências da Saúde, Macapá, AP, Brazil.

**Keywords:** Border Health, Border Areas, Qualitative Research, Mining, Coronavirus Infections, Salud Fronteriza, Áreas Fronterizas, Investigación Cualitativa, Minería, Infecciones por Coronavirus, Saúde na Fronteira, Áreas de Fronteira, Pesquisa Qualitativa, Mineração, Infecções por Coronavírus

## Abstract

**Objective:**

To analyze the conceptions about COVID-19 among Brazilians who carry out commuting to work in clandestine mines located on the borders between Brazil, French Guiana and Suriname.

**Method:**

This is qualitative research, from an analytical perspective, based on Social Representation Theory. Semi-structured, audio-recorded interviews were carried out with 10 Brazilians who experience work routine in clandestine mining on the border between Brazil, French Guiana and Suriname.

**Results:**

Two analytical categories emerged: “The disease of otherness”; and “Health access dimension”.

**Conclusion:**

Disease severity was attributed to another or a human body organ, and not to individuals as a whole. Access to health services was established on issues of inequity, violence and illegal practices. The nature of a transient population, which carries out commuting and informal and clandestine work, demonstrates vulnerability to COVID-19 and a lower propensity to receive care.

## RESUMO

**Objetivo:**

Analisar as concepções acerca da covid-19 entre brasileiros que realizam migração pendular para trabalhar em garimpos clandestinos situados nas fronteiras entre Brasil, Guiana Francesa e Suriname.

**Método:**

Pesquisa qualitativa, em uma perspectiva analítica, alicerçada na Teoria das Representações Sociais. Foram realizadas entrevistas semiestruturadas, áudiogravadas com 10 brasileiros que vivenciam a rotina de trabalho em garimpos clandestinos na fronteira entre Brasil, Guiana Francesa e Suriname.

**Resultados:**

Foram designadas duas categorias analíticas: “A doença da alteridade”; e “Dimensão do acesso à saúde”.

**Conclusão:**

A gravidade da doença foi atribuída ao outro ou a um órgão do corpo humano, e não ao indivíduo como um todo. O acesso aos serviços de saúde instituiu-se em questões de iniquidade, violência e práticas ilegais. O caráter de população transitória, que realiza migração pendular e de trabalho informal e clandestino, demonstra vulnerabilidade à covid-19 e menor propensão a receber cuidados.

## RESUMEN

**Objetivo:**

Analizar las concepciones sobre el COVID-19 entre los brasileños que realizan desplazamientos para trabajar en minas clandestinas ubicadas en las fronteras entre Brasil, Guayana Francesa y Surinam.

**Método:**

Investigación cualitativa, desde una perspectiva analítica, basada en la Teoría de las Representaciones Sociales. Se realizaron entrevistas semiestructuradas y grabadas en audio a 10 brasileños que viven la rutina de trabajo en minas clandestinas en la frontera entre Brasil, Guayana Francesa y Surinam.

**Resultados:**

Se designaron dos categorías analíticas: “La enfermedad de la alteridad”; y “Dimensiones del acceso a la salud”.

**Conclusión:**

La gravedad de la enfermedad se atribuyó a otro o a un órgano del cuerpo humano, y no al individuo en su conjunto. El acceso a los servicios de salud se estableció sobre temas de inequidad, violencia y prácticas ilegales. La naturaleza de la población transitoria, que realiza desplazamientos y trabajos informales y clandestinos, demuestra vulnerabilidad al COVID-19 y una menor propensión a recibir atención.

## INTRODUCTION

The Brazilian border strip with French Guiana and Suriname has several sociocultural and economic specificities, highlighting an intrinsic relationship with clandestine mining and trade in *Euros* with French Guiana^(1)^. In this regard, it is estimated that there are around eight thousand miners working in around 600 clandestine gold mining sites in the region^(2)^.

This scenario favors the commuting flow of people, which also benefits the worsening of health and disease conditions^(3)^. It is worth highlighting that twin cities and cross-border crossing areas are more vulnerable to various health problems, such as HIV infection, tuberculosis and leprosy^(3)^. It is also observed that the Oiapoque-Tumucumaque border sub-region is one of the most critical areas on the Brazilian border for these diseases and also for health care, where there is a lack of access to goods and services^(4,5)^.

The migratory movement in search of better living conditions, associated with high cross-border mobility, precarious housing and work conditions, difficulty in accessing the region by health teams, shortage of qualified professionals and persistent incursion of miners into the forest, favors the rapid spread of diseases^(1,3,5)^.

This large displacement and transit of people, added to the enormous geographic extension of the Amazon region and accentuated social inequalities and ethnic-cultural pluralities, make it difficult to spatially analyze the vulnerabilities of subjects who are in mining areas, making it difficult to analyze and understand the processes that produce illness and health recovery in the region^(3,6)^.

In addition to these difficulties, emerging infectious diseases represent a threat to public health^(7)^. Thus, the new coronavirus pandemic constitutes a major challenge for the Amazon border region, since usual strategies to contain the spread of the pandemic, such as social distancing and case isolation, were presented in a context of clandestine activities and high population flow. Therefore, numerous conditions, including social, economic issues and community behavior, can have an impact on the measures adopted, influencing the spread and mortality of SARS-CoV-2.

It is observed that the mines are part of a concrete social order built by miners themselves^(8)^. There is no geographical delimitation for the exercise of mining activities, as the borders defined by the State are not obeyed from the perspective of mining. Miners migrate from one country to another without following established rules^(9)^.

In this context, the objective was to analyze the conceptions about COVID-19 among Brazilians who carry out commuting to work in clandestine mines located on the borders between Brazil, French Guiana and Suriname. To this end, the lenses of Social Representation Theory (SRT) were used^(10)^. SRT deals with social knowledge production, especially those that are produced in daily life. These beliefs are socially elaborated and shared with the purpose of constructing and interpreting a reality common to a social group, and are strongly linked to the experience from which they originated and, above all, to the contexts and conditions in which they are placed^(10)^. From this perspective, it is expected that the results of this study may raise questions about the understanding of COVID-19 by this population, providing perspectives for constructing strategies to contain the spread of the disease in the context of the Amazon border and surrounding countries.

## METHOD

### Design

This is research with a qualitative approach, in an analytical perspective based on SRT, based on the theoretical framework proposed by Moscovici^(10)^.

### Place

The study was carried out in the municipality of Oiapoque, Amapá (AP), Brazil, twin city of Saint Georges, in French Guiana. These countries border Suriname. The small city has around 25 thousand inhabitants. It is located in the extreme north of Brazil and serves as logistical support for clandestine mining activities in the region.

### Period

Semi-structured interviews took place from July to December 2020.

### Population

The study included 10 Brazilians, over 18 years of age, who carry out commuting to work in clandestine mines on the border between Brazil, French Guiana and Suriname, with a logistical support point in the city of Oiapoque, AP. Recruitment was carried out using the snowball technique, using exponential sampling, used to reach difficult-to-reach groups^(11)^. All people approached agreed to participate in the study. The sample size was determined by identifying the data saturation point, when no new themes were recorded in the interviews^(12)^.

### Data Collection

Semi-structured interviews were carried out, lasting an average of 45 minutes. A single interview was carried out with each participant, through social applications or radio, the main means of communication in mines. Contact between those involved cannot be made in person, due to the pandemic context experienced during the data collection period. Access to participants was made possible through research networks previously built in Oiapoque by the main researchers of the study, who lived in the city for four years, due to their work activities. The following guiding questions were asked: 1) what do you understand by the COVID-19 (coronavirus) pandemic? and 2) if you need health care, what actions, people and places do you turn to? These questions sought to identify the representations anchored around the new coronavirus pandemic and identify access to health for this population.

A pilot study was carried out with one participant to verify the clarity and understanding of the guiding questions, who also indicated his consent to the research. There was no need for adjustments. The data collected in the pilot study were excluded from the research.

### Data Analysis and Treatment

The material was subjected to thematic content analysis, described below. The pre-analysis stage consisted of a first contact through material skimming, followed by reflective reading to organize the recording and context units, allowing guidance and direction in the analysis of impressions. In the material exploration stage, researchers carried out the distribution and categorization scheme, and, subsequently, organization of trends and other characteristic determinations of the phenomenon investigated.

Statement categorization was carried out by two researchers, with different backgrounds, from the field of health and human sciences, looking for points of convergence and divergence in analysis. In the treatment and interpretation of results obtained, the content underlying what was being manifested was coded, articulating the objectives of the study with the trends and theoretical content covered^(13)^. The entire coding process is described in Chart 1.

**Chart 1:** Research data coding – Macapá, AP, Brazil, 2023.

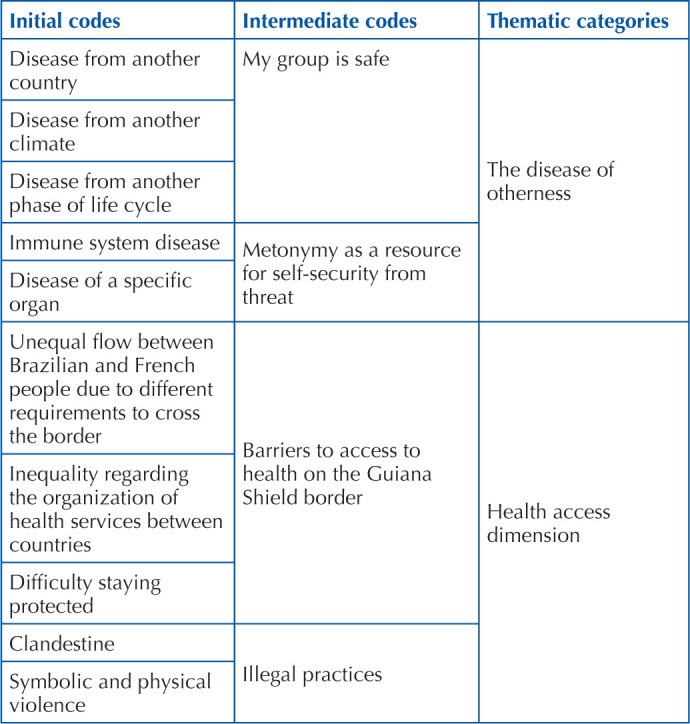

Source: collection data, 2023.

Categories’ reflection and analysis were based on a diligent perspective of understandings engendered by native categories, with actors’ speech having a fundamental weight in interpreting the pandemic’s social representations and miners’ access to health services. However, it is noteworthy that, to understand the meaning cores, narrative analysis is not enough. It is necessary to bring together the social and historical conditions of production and circulation of conceptions shared by the group that gave rise to them. In this process, understanding the context in which people are led to react to a stimulus provides elements for understanding the processes of formulating their representations^(10)^.

To identify the conceptions attributed by actors, it was necessary to resort to two SRT concepts that helped in the process of understanding miners’ knowledge about COVID-19: objectification and anchoring. Objectification is the figurative phase, the result of the ability that thought and language have to materialize the abstract into a new concept based on existing individual records. It is the path through which representations acquire materiality. Anchoring is the transformation of something strange and disturbing, which intrigues us, into our particular system of categories, comparing it with paradigms of a category that we believe to be appropriate^(10)^.

### Ethical Aspects

This study followed Resolution 466/2012 of the Brazilian National Research Council, obtaining favorable Opinion 4,085,017, issued by the Research Ethics Committee of the *Universidade Federal do Amapá* in 2020. Participants in the research and pilot study expressed consent to participate in the study through recorded audio. *Consolidated Criteria for Reporting Qualitative Research (COREQ)* guidelines were also used to comply with methodological rigor. To guarantee participant secrecy and confidentiality, interviews were identified using the letter I (interviewed), followed by random numbers assigned by the researchers.

## RESULTS

Participants were mostly male, black and brown, aged between 20 and 30 years old, born in the North region, with incomplete high school, living in a stable union and carrying out occupational activities directly related to gold extraction. Informative means for acquiring knowledge about COVID-19 were predominantly social networks, through cell phone applications, friends and relatives, and television news. COVID-19, by miners, was materialized through access to informative videos, shared by social media groups, mainly WhatsApp®.

As a result, two categories were identified: “The disease of otherness”; and “Health access dimension”. The first nucleus addressed the disease understanding and representations from actors’ perspective. The second verified questions about the extent of access to health services and the pandemic repercussions in the context of borders and clandestinity.

### The Disease of Otherness

COVID-19 had its representation anchored to its external origin and characteristics that are different from those observed in the mining environment. From this perspective, it appears that COVID-19 is portrayed in the mining environment as a disease that affects other realities than those experienced by miners: *COVID-19, for me, is a virus that was created in China, and that people who have this virus have difficulty breathing, and it spreads to people mainly in the cold* (I2).

It was verified, among those interviewed, that the association of infection by the virus was anchored as a risk to people, with distinct and external properties to which miners are exposed, whose formation is mostly made up of young adults, located within the tropical forest, in hot and humid climate: *Older adults, sick people, who tend to have worse complications due to their low immunity; people who have good immunity can survive with access to treatment* (I1). Thus, it appears that the representation was anchored in otherness, materialized in the other; it is the “cold” disease that affects “older adults” and “people with low immunity”, and is not something considered threatening to miners.

It was also possible to identify, in speeches, the representation of the disease’s ontological model. The disease is portrayed as a “being”, “cursed”, that invades and degenerates the body. *It’s a damn disease, right? It’s a virus that’s all over the world, right? And it causes serious lung disease* (I3). These are locating representations that bring security to individuals. In fact, it is reassuring to know that the disease is related to an organ, a part of the body, in this case, the lung and not the self as an individual.

### Health Access Dimension

Mobility restrictions, increasingly severe from France to Brazil, were the first mishap reported by residents of these border areas, which was later enhanced by other methods and effects to deal with a pandemic.

*The problem is that there is a very large flow of French here. We can’t go there* [French Guiana], *but they come here easily* (I6). *The Federal Government did not allow people to cross to Brazil because of this disease. There I couldn’t sign the expulsion paper, so we went back to French Guiana; then the Federal Government decided to bring us to Oiapoque because of the way the gendarmes* [French Guiana police] *treated us; then they sent us back to Brazil* (I4).

It is important to highlight that these restrictions on mobility between Brazil, French Guiana and Suriname are part of an overview that goes beyond pandemic issues, since there is a large number of Brazilians who try to make a living in French territory, either by paying for work in *Euros*, whose currency value is higher than the *Real*, or by trying to make a living in illegal gold mines located in the territory French. This reflects tough measures by French authorities regarding the requirement for documents to allow the presence of Brazilians in their territory, which is not required of the French by Brazilian authorities.

It can be seen, among the statements, that the condition of clandestinity exacerbates access difficulties, exposing this population to health problems that go beyond the conditions imposed by the COVID-19 pandemic, such as experiences of physical violence and psychological distress.

*The police arrested us there in Suriname. The police there* [French Guiana] *made us walk from Suriname to the bridge. When we arrived here at the bridge, there were people feeling sick, 22 people with swollen, injured feet, horrible, horrible. The Federal Police gave water and snacks, and said that the police there* [gendarmes] *couldn’t do that, that, even without cars coming, they should find a way to bring them, they couldn’t bring them by walking all that distance* (I6).

Participants were aware of their vulnerability in relation to the COVID-19 pandemic, and outlined perceptions about structural health problems at the border. It is also observed, from interviewees’ speeches, knowledge about ways to prevent the disease, whether by using masks or verbalizing the importance of social isolation. However, the lack of negotiating power regarding the use of Personal Protective Equipment (PPE) with the French police is also revealed, given the clandestinity condition.

*Everyone has to be aware that they have to stay there and we have to stay here, because this flow is as bad for them as it is for us. Their advantage is that the road to Cayenne is all paved. It takes 4 hours at most, the car goes slowly and arrives in Cayenne, and we don’t* (I6). *It’s been about 10 days since I arrived from the mining site in front of Suriname. There no one takes care of themselves, no one uses a mask or gel, there we go to work. The gendarmes who caught us weren’t wearing masks, so they brought us to the city* [Saint George], *everyone without masks* (I8). *There* [French Guiana] *he is fined. If they catch him on the street, it’s 135 euros. I know that, in Saint George, there were 3 serious cases. Do you know what they did? The plane came quickly and took us to Cayenne. They have support, they have support. Their service works* (I7). *Not even there at the barrier* [Grand Rochè Waterfall] *there was no mask. In Saint George, it was isolated, everything was locked* (I4).

The topic of access to services was also raised by participants. In this regard, it is important to highlight that access to health services is not achieved by miners, mainly because they do not have the freedom to choose how to use these services, mainly due to the clandestinity they are in.

*I was talking to her yesterday, and she was feeling some pain and stuff. And there’s no way to go to the hospital, because, to cross, it’s really bad, there’s a lot of police. And we’re in a little place close to Suriname, but it’s illegal, right? We arrived now and we can’t get down, because there are a lot of police, and everyone is very scared, because there is a suspicious case here, and then you can’t go to the doctor, you can’t cross, you have to die here* (I7).

## DISCUSSION

Conceptions about COVID-19 by miners in materializing the disease as otherness, of older adults, of the cold, of those with low immunity permeate unique models of representation that, historically, were also anchored in the understanding of other epidemic diseases throughout history as an individual’s defense against the threat presented^(14)^. For instance, when syphilis ravaged Europe during the 15^th^ century, it was categorized as “the French disease for the English, the *morbus Germanicus* for the Parisians, the disease of Naples for the Florentines, and the Chinese disease for the Japanese”^(15)^. In line with this perspective, studies on cross-cultural representations of HIV infection also demonstrated perceptions of relational models of the disease, where it was attributed to non-hegemonic practices of other groups, such as sexual practices between people of the same sex^(16)^.

Thus, contrary to what was anchored by miners, it can be seen in the literature that most hospital admissions for serious cases of COVID-19 in Brazil occurred among young adults between 20 and 59 years old, making it possible to identify a high mortality rate due to COVID-19 in the North region (93.7/100 thousand inhabitants), specifically in the state of AP (101/100 thousand inhabitants)^(17)^. Regarding this aspect, it is noteworthy that, when comparing the North and South regions, it is clear that, even though the latter has a higher percentage of older population, mortality from COVID-19 is higher in the North region, which denounces health structure fragility of this Amazon region^(4)^ and the abysmal inequality that exists between Brazilian regions.

These disparities indicate that social aspects can greatly influence the course of the disease, since the number of deaths is higher in populations living below the poverty line^(18)^. In this regard, it is also noteworthy that predominantly rural areas in the Amazon region, with a low and medium Human Development Index and precarious access to treated water, sewage disposal and electricity have a high risk of sustained transmission of COVID-19^(18)^.

Participants’ conceptions also attributed the cure of the disease to people with “good immunity”, who “can survive with access to treatment”. This way of meaning the disease is similar to the disease’s functional and relational model^(14)^. In this model of disease representation, each individual belongs to what we today call a type, characterized by the unique particularity of a balance and also by predisposition to a certain imbalance; in this case, advanced age, low immunity, or living in locations with exposure to intense cold.

Perception, therefore, depends on the characteristics of sick people or places to which they are exposed. Even if refutable, it can also be explained by what is called focusing^(10)^, i.e., a way of assimilating information by attributing attention and relevance to certain subjects instead of other aspects.

Men are possessed by an instinctive fear of powers that they cannot control, and they try to compensate for this impotence in an imaginative way^(10)^, anchoring their representations in places of security of the self. In this regard, the disease appears as belonging to otherness, to the other^(14)^. Thus, despite indicators pointing to a high incidence in the state of AP, there is prospect of creating an imaginary representation of a safe environment for individuals’ health in terms of the development of severe forms of the disease.

The disease’s ontological model can also be seen in the statements. This model designates the task of delegating the virus’ harmful effects to a human body organ^(14)^. The disease, in this way, no longer has relations with sick people, but with a part of their body.

Another highlight of participants’ speeches was health access dimension. Regarding this aspect, it is important to highlight that this border usually has an unequal flow and transit dynamic between Brazilian and French people^(1)^. French people have no legal restrictions to come and go to Brazilian territory, while Brazilian people need a visa to have legal access to French Guiana. Mobility on the border has always been, for these populations, a point of intense conflicts and disputes^(19)^, which, added to issues related to the pandemic, was presented in the verbalization of Brazilian people’s desires for control and restriction of French people’s entry into territory Brazilian.

Thus, the condition of clandestinity imposes several obstacles to access to health services^(1)^. Added to clandestinity are the distance from health establishments, the absence of mobile Intensive Care Units that can travel to remote locations in the Amazon, or even the impossibility of identifying miners as citizens of another country, which is not their country of origin^(5)^.

From this perspective, this discussion does not constitute a defense of illegal practices, but presents tensions regarding the right to health that permeates other rights, such as the freedom to come and go, decent housing, education and food, even observing that these aspects clash with the conceptions and ways of each country dealing with the health and other rights of those who cross their borders without authorization. It is thus demonstrated that mining in the Amazon and the commuting mobility resulting from it cannot be reduced to a police issue, through the use of coercion and force, but permeate the guarantee of other rights so that access to health is full.

The study has limitations, since collection was carried out via radio and social applications, which made it impossible to observe daily life in mining *currutelas* (population centers located in mining areas) themselves. However, despite these limitations regarding coronavirus contagion prevention, this study made it possible to elucidate representations about the pandemic in a context of social vulnerability. In this regard, this study elucidated the pandemic’s social representations based on miners who commute between bordering countries (Brazil, French Guiana and Suriname). Based on these concepts, it becomes possible to identify strategies to contain the spread of the disease in the Amazon border and surrounding countries, such as itinerant health units in places of rest and logistical support.

## CONCLUSION

Representations were anchored in otherness and locating meanings. Disease severity was attributed as specific to another or a human body organ, and not to individuals as a whole. Access to health services was established on issues of inequities, violence and illegal practices. The nature of a transient population, which carries out commuting and informal and clandestine work, demonstrates vulnerability to COVID-19 and a lower propensity to receive care.
